# Development and Evaluation of Clinical Practice Guideline for Delirium in Long-Term Care

**DOI:** 10.3390/ijerph17218255

**Published:** 2020-11-09

**Authors:** Eunhye Jeong, Jinkyung Park, Sung Ok Chang

**Affiliations:** 1 College of Nursing, Korea University, Seoul 02841, Korea; preaquaty@korea.ac.kr (E.J.); carpe@korea.ac.kr (J.P.); 2 Transdisciplinary Major in Learning Health Systems, Graduate School, Korea University, Seoul 02841, Korea

**Keywords:** delirium, aged, guideline, long-term care, nursing home

## Abstract

Delirium is highly prevalent and leads to several bad outcomes for older long-term care (LTC) residents. For a more successful translation of delirium knowledge, Clinical Practice Guidelines (CPGs) tailored to LTC should be developed and applied based on the understanding of the barriers to implementation. This study was conducted to develop a CPG for delirium in LTC and to determine the barriers perceived by healthcare professionals related to the implementation of the CPG. We followed a structured, evidence- and theory-based procedure during the development process. After a systematic search, quality appraisal, and selection for eligible up-to-date CPGs for delirium, the recommendations applicable to the LTC were drafted, evaluated, and confirmed by an external group of experts. To evaluate the barriers to guideline uptake from the users’ perspectives, semi-structured interviews were conducted which resulted in four major themes: (1) a lack of resources, (2) a tendency to follow mindlines rather than guidelines, (3) passive attitudes, and (4) misunderstanding delirium care in LTC. To minimize adverse prognoses through prompt delirium care, the implementation of a CPG with an approach that comprehensively considers various barriers at the system, practice, healthcare professional, and patients/family levels is necessary.

## 1. Introduction

Delirium is a neuropsychiatric syndrome that manifests sudden change and fluctuation of attention, consciousness, and cognitive function [1]. It is known that older adults are particularly vulnerable to delirium due to being more likely to present representative risk factors of delirium such as older age, dementia, and reduced mobility [2]. In long-term care (LTC), where the older adult population is dominant, the prevalence of delirium reaches up to 70% and is even higher, up to 89%, in older adults with dementia [3,4]. Notably, delirium in this population is considered a medical emergency due to its negative prognosis caused by the failure to detect it early [5,6].

Delirium is often overlooked in various clinical settings, particularly in older adults [3]. There are several reasons why delirium screenings frequently fail in this population: the difficulty of differential diagnosis from delirium superimposed on dementia (DSD) or depression [7,8], the higher prevalence of persistent delirium and subsyndromal delirium (SSD) [9,10], and hypoactive delirium characterized by quiet and subtle symptoms [11]. In addition, healthcare professionals’ lack of delirium knowledge has been a major cause of underdiagnosis [12].

In LTC, unlike hospitals, the presence of an actual physician is limited, compelling all healthcare professionals who are at the bedside to play the central role in delirium management [13]. Additionally, in LTC, most residents are older adults who tend to stay for a relatively long time under the major purpose of conservative rather than active treatment, allowing them to be cared for from more long-term perspectives. These distinct contexts of LTC in many countries, as well as in Korea, make it difficult to directly apply existing Clinical Practice Guidelines (CPGs) for delirium. Furthermore, based on these features, the specific barriers related to the implementation of the CPG in this setting should be identified for more effective uptake of the CPG [14].

For a more successful translation of delirium knowledge, CPGs tailored to LTC should be applied based on the understanding of the barriers to implementation of CPGs [14]. Thus far, several delirium CPGs have been developed. However, to the best of our knowledge, there is no available CPG for delirium in LTC.

Thus, the aims of this study were set as following: (1) to develop and implement evidence-based CPGs for delirium specific to LTC and (2) to determine the barriers perceived by healthcare professionals related to the implementation of the CPGs. In this study, key questions for the development and appraisal of CPGs for delirium were written in the form of PICO (P: patient, I: intervention, C: comparison, O: outcome). “Patient” was set as older adults who are not limited to a specific treatment environment or specific patient group, “intervention” as a strategy for delirium management that includes three sub-domains: prevention, early detection, and intervention, “comparator” as conventional routine treatment, and “outcome” as usefulness and effectiveness. The key PICO questions are as follows.

(1) What strategy is recommended for preventing delirium in older adults (concerning high-risk group management)?
(2) What strategy is recommended for early detection of delirium in older adults?
(3) What strategy is recommended for the intervention of delirium in older adults?

## 2. Materials and Methods

### 2.1. Development of the CPG

This study carried out an overall process based on the detailed steps of CAN-IMPLEMENT, which was developed by the Canadian Guideline Adaptation Study Group, using the Ottawa Model for Research Utilization (OMRU) and the Knowledge to Action (KTA) frameworks [14,15].

#### 2.1.1. Search and Screen

For discovering relevant CPGs, this study conducted a systematic search for CPGs with high-level evidence. The CAN-IMPLEMENT recommends using CPG search databases, country-specific databases, and the websites of guideline development organizations to collect evidence-based CPGs. Therefore, we utilized various CPG sources, including the websites of the Guidelines International Network (G-I-N), New Zealand Guideline Group (NZGG), National Institute for Health and Care Excellence (NICE), Scottish Intercollegiate Guidelines Network (SIGN), and Canadian Medical Association, as well as general databases including MEDLINE, CINAHL, PsycArticles, and Korean databases. Search terms related to “guideline” and “delirium” were used, but terms related to target patients (older adults), setting (LTC), or outcomes were not used, in order to obtain highly sensitive results. For a more extensive data search, the reference list of related studies and a Google search were also used. The CPGs were searched for and screened by two authors independently (E.J. and J.P.). The process was conducted in March 2019, and it was confirmed that there were no newly added CPGs in a repeat search following the same process in March 2020.

The selection criteria for CPGs were: (1) developed within the last five years and (2) published in English or Korean. The exclusion criteria were: (1) developed by an individual, (2) specific target setting other than LTC, such as an intensive care unit or emergency department, (3) specific target population other than older adults, (4) not an original CPG, and (5) quality assessment results of less than 50% for the Appraisal of Guidelines for Research and Evaluation II (AGREE II) [16].

#### 2.1.2. Quality Assessment and Selection

In this step, to evaluate the quality of the CPGs, we used AGREE II, which is an evaluation tool for CPGs. AGREE II was developed with the aim of standardizing CPGs with different quality levels and measuring methodological rigor and transparency [16]. This tool consists of 23 items in six areas: scope and purpose, stakeholder involvement, rigor of development, clarity of presentation, applicability, and editorial independence. The CPGs were assessed and selected by two authors (E.J. and J.P.) independently.

#### 2.1.3. Draft, Revise and Endorse Recommendations

For drafting recommendations, the core information of the selected CPGs was extracted using a predetermined Excel form (Table S1). In this study, recommendations were adapted based on the three PICO questions that were set in advance, and the level of evidence for each recommendation was extracted [17]. Through comparison of the detailed recommendations of each selected CPGs, only those mentioned in two or more CPGs were selected and then constructed according to the PICO questions.

For revision and confirmation of the draft, an external review was conducted by a group consisting of four experts (two professors qualified in geriatric care, a manager of LTC, and an experienced healthcare professional working in LTC for more than 10 years). For the overall evaluation of the drafted CPG, we used the questionnaire for assessments of CPGs [18], which consists of four domains: (1) guideline quality (need for a guideline on delirium, clearness of drafted CPG), (2) applicability (agreement, suitability for older LTC residents, predicted benefits and harms, acceptability of presented options, the possibility to be supported by most colleagues, expected effects), (3) acceptability (rigidity to apply, need for reorganization of services, technical concerns, economic aspects), and (4) comparative value (effectiveness, better use of resources). Additionally, each recommendation of the draft was evaluated in three domains: degree of agreement, applicability, and clinical importance, and an item was selected as significant when more than 80% agreement on it was reached.

### 2.2. Post-Interviews

To identify the barriers to implementation perceived by healthcare professionals, we conducted semi-structured interviews after the four-week implementation of the developed CPG in two LTC settings in South Korea. The developed CPG has been implemented by all staff including managers, nurses, social workers, physical therapists, and health assistants since May 2019. The healthcare professionals with more than three years of clinical experience, who agreed to be interviewed, were invited to participate. They were asked questions about the barriers or difficulties perceived during the implementation of the CPG. All interviews were audio-recorded and transcribed. For thematic analysis, the following six phases were applied: (1) familiarizing yourself with your data, (2) generating initial codes, (3) searching for themes, (4) reviewing themes, (5) defining and naming themes, and (6) producing the report [19].

### 2.3. Ethical Considerations

This study was carried out as part of the project “Development and Effectiveness of Delirium Education Program for LTC Healthcare Providers” and approved by the Ethical Committee at the university the authors belong to (KUIRB-2019-0038-01).

## 3. Results

### 3.1. Development of the CPG

#### 3.1.1. Search and Screen

**Figure 1:**
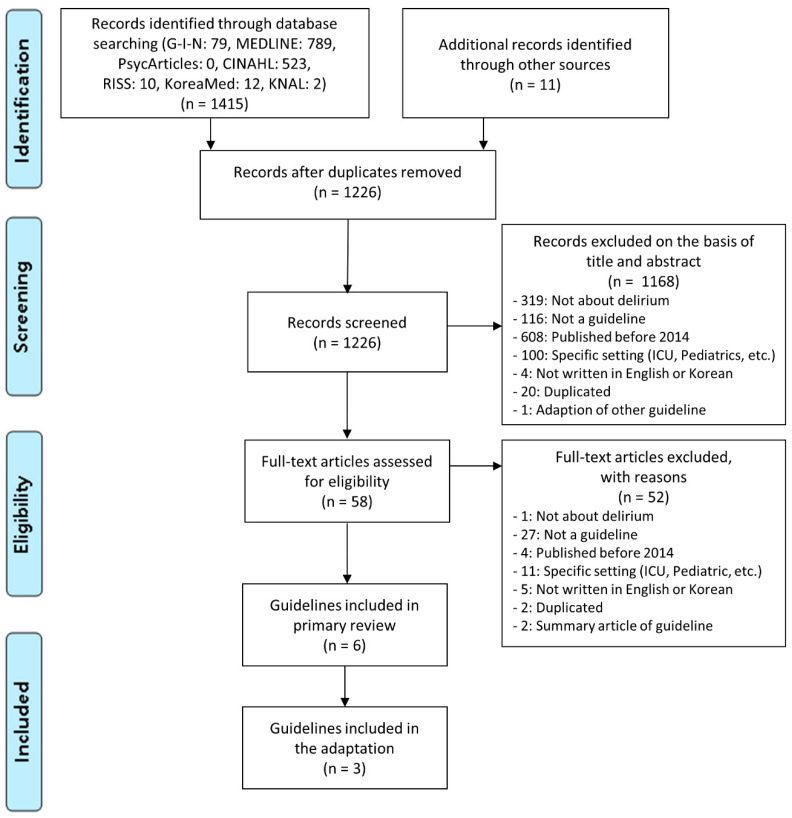
Flow chart of the search for eligible CPGs. CPG, Clinical practice guideline; G-I-N, Guidelines International Network; KNAL, Korean National Assembly Library.

Figure 1 provides a detailed search and selection flow.

#### 3.1.2. Quality Assessment and Selection

From the six CPGs included in the primary review, three that were evaluated at above 50% in quality assessment using AGREE II were included in the final review [17,20,21]. The characteristics of the included CPGs are presented in Table 1.

#### 3.1.3. Draft, Revise, and Endorse Recommendations

Out of a total of 68 recommendations (Registered Nurses’ Association of Ontario (RNAO) = 16, NICE = 27, and SIGN = 25), after the exclusion of inapplicable ones (e.g., not possible to perform in LTC) and the integration of duplicated ones, 17 were finally selected as recommendations applicable to the clinical environment of LTC.

As a result of an external review by the expert panel, minor modifications, such as a change in wording or expressions for readability, were made. The recommendations with scores of 80% in three domains (degree of agreement, applicability, and clinical importance) were included in the final CPG. Overall evaluation using the questionnaire for assessments of CPGs also reached 80%. The final CPGs are presented in Table 2.

### 3.2. Post Interviews

Ten healthcare professionals participated. They were all women, consisting of two managers, five registered nurses, three health assistants, with an average of 15.1 ± 10.7 years of clinical experience (range: 6 to 37). Face-to-face interviews, averaging 29 min in length (range: 12 to 61), were conducted between June to August 2019. A thematic analysis revealed four major themes (Table 3).

## 4. Discussion

This study was conducted to develop a CPG for delirium in Korean LTC settings and to determine the barriers to its actual implementation. Although many CPGs for delirium have been developed globally, it is difficult to apply them directly to LTC because of different conditions, context, patient populations, and barriers related to implementation [22]. This study provided the latest evidence-based resources regarding delirium care for older LTC residents and also identified the possible barriers to consider for more effective implementation of CPG for delirium.

### 4.1. Recommendations of the CPG

#### 4.1.1. Domain 1. Prevention through the Management of Risk Factors

For delirium prevention in LTC, the screening of delirium risk factors for all older adults at admission and whenever there is a change in their condition is essential (Recommendation 1) [17,20]. Since advanced age (>65 years) is a leading delirium risk factor, all older LTC residents should be considered high risk [20]. Furthermore, it should be taken into account that this population is also likely to have additional representative predisposing factors of delirium, such as multiple comorbidities, polypharmacy, and underlying cognitive impairment (Recommendation 1) [17,20].

Consequently, in LTC, all older residents should be routinely provided tailored strategies for delirium prevention via the continuous implementation of a package of multiple non-pharmacological approaches based on each individual’s risk factors, undertaken through the collaboration of a multi-disciplinary team (Recommendation 2) [17,20,21]. In addition, pharmacological risk reduction should also be applied (Recommendation 3). Notably, a systematic review of randomized controlled trials reported that a pharmacist-led medication review for LTC residents had a significant impact on decreasing delirium incidence [23]. Nevertheless, since pharmacists in this setting are in reality very limited in number, a detailed protocol for medical reviews by healthcare providers, such as nurses, who are on the front line of delirium care in this setting, should be further developed and disseminated.

#### 4.1.2. Domain 2. Early Detection

For early detection, which is the most important factor in delirium care, it is essential to use a valid tool for screening delirium at least once a day (Recommendation 4) [17,20]. In particular, if there is an acute change or fluctuating course of cognitive functions, attention, or alertness, it should be detected using a screening tool immediately (Recommendation 4) [17,20,21]. It should also be noted that, in LTC where an older adult population is dominant, the differentiation of delirium from dementia and depression is particularly important (Recommendation 6) [17,20]. Additionally, healthcare providers should pay special attention to hypoactive delirium, which is more prevalent in this population (Recommendation 5) [20]. If delirium is suspected, it should be diagnosed by a qualified healthcare professional in a referred hospital or by attending physicians (a psychiatrist or a neurologist) who visit regularly (Recommendation 7) [17,20,21]. After delirium is diagnosed, it should be documented in the patient’s medical record and the patient and family notified. Their opinions should be respected concerning a preference for conservative rather than active management (Recommendation 8) [17,20,21].

#### 4.1.3. Domain 3. Intervention

Healthcare providers should be aware that in most cases delirium has multiple causes and that they should systematically identify the underlying causes (Recommendation 9) [17,20,21]. When delirium is suspected or diagnosed, non-pharmacological treatment should be first implemented through multidisciplinary cooperation (Recommendation 11). The evidence of pharmacological intervention for delirium has not yet been clarified, but in the following situations—1. application of non-pharmacological intervention of delirium was ineffective, 2. the patient is distressed, or 3. there is a possibility of harming others or self)—medications for relieving delirium symptoms can be considered (Recommendation 13) [20]. Additionally, analgesics should be actively recommended for effective pain management since pain is one of the leading causes of delirium (Recommendation 14) [17].

In older adults who have not recovered after the intervention, there may be an existing cognitive impairment that may or may not have been discovered; hence, additional cognitive and physical function evaluations should be conducted (Recommendation 16) [17,20,21]. Throughout the process of delirium care, referring the patient to hospitals for appropriate follow-up by qualified healthcare professionals should be considered, if possible (Recommendation 17) [17,20,21].

Although specific recommendations about SSD, persistent delirium, and DSD were limited in the included CPGs due to a lack of evidence, healthcare providers in LTC should consider those conditions when delirium is not relieved or lasts a relatively long time [24]. Usually, the onset and course of most delirium episodes are acute and short-term, ranging from hours to days. However, the prevalence of persistent delirium that lasts several weeks to months is significantly higher in older adults [25] and even higher when including SSD, which is a partial delirium that does not fully meet the diagnostic criteria [9]. Especially in the older adults with dementia population, persistent delirium or SSD is more prevalent and has a worse prognosis [26,27]. As such, further study is necessary to create evidence-based recommendations on prevalent forms of delirium for older LTC residents, such as DSD or SSD.

In conclusion, it would be an effective strategy to apply the most important and basic recommendations (Recommendation 2, 4, 11) as a central framework for delirium management and implement the remaining recommendations together. First, preferentially, a valid tool for delirium screening should be successfully embedded in clinical practice. Among the widely used, validated, and easy-to-use (required time <5 min) tools for delirium detection, such as the 4 ‘A’ test (4AT) or Nursing delirium screening scale [28,29], an appropriate tool should be selected. Notably, the 4AT could be the first option, since SIGN recommends the tool be considered for use in community or other settings [21]. Second, an individualized care plan for delirium prevention and treatment should be developed and implemented at admission and continuously updated on a regular basis (e.g., weekly) by specific strategies suitable to each LTC context.

### 4.2. Barriers to Implementation of CPG

There have been some studies on barriers for delirium care conducted in the intensive care unit or palliative care [30,31], but studies in LTC settings are lacking. In this study, at the system level, a lack of resources and opportunities for delirium education was identified as a barrier to the implementation of CPG, which is in line with previous studies [30,31]. The healthcare professionals, in this study, needed delirium education and delirium screening tools applicable to LTC. Considering the limited presence of doctors who can immediately diagnosis and manage delirium in this setting, support strategies, such as the provision of more practical and detailed delirium education, and valid and simple tools for delirium detection, should be provided in the system level.

At the practice level, we identified the tendency of healthcare providers in LTC to rely on their own knowledge rather than the evidence-based guidelines as a barrier to guideline uptake. They chose to follow their own mindline rather than to follow the latest evidence-based guidelines. They regarded themselves as already familiar enough with all residents, having had no specific delirium concerns for many years. The problem revealed in this study was that managers of LTC settings, as well as other practitioners, also agreed to follow this tacit knowledge. A previous study also reported on nonreceptive organizational culture to evidence-based practice in LTC [32]. Despite the clear evidence of the benefits to LTC residents of evidence-based CPGs, their adoption in practice in LTC has remained slow and sporadic [33]. The LTC staffs, including especially the managers and administrators, need to understand why delirium practice should be improved, how the evidence-based CPG will improve practice, and what differences will be made in residents through the implementation of the CPG.

At the healthcare professionals level, the practitioners’ limited knowledge or experiences and passive attitude regarding delirium were identified as barriers to guideline uptake. Limited delirium knowledge can lead to lower confidence and passive attitudes regarding delirium care [34]. The interviewed healthcare professionals stated that there have been few delirium cases observed for many years, yet it should be noted that the reason why they observed few delirium cases might be their lack of delirium detection skills. In fact, a systematic review reported the prevalence of delirium in LTC is much lower in retrospective studies (1.75–2.3%) than overall (up to 70%) due to the possibility of many missed delirium cases by practitioners [3]. Delirium education for LTC practitioners, therefore, should be accompanied by specific strategies or tests to detect the main features of delirium that are likely to be mistaken as underlying cognitive disorders, such as how to detect inattention, altered alertness, or disorganized thinking.

At the patients/families’ level, their misunderstanding that delirium care is always an aggressive treatment was identified as a possible barrier to implementation of the CPG. Since delirium education for patients/families is recommended (Recommendation 12) [17,20], they should be informed that the main purpose of delirium care is to reduce the unfavorable prognoses of patients, such as sores, falls, and cognitive decline, and that it is essential in terms of securing the comfort and relief of suffering [5,6,35]. For improving the patients/families’ understanding, real cases of patients with experience of delirium could be helpful [17,35]. Further, educational materials should be separately developed for the residents and families based on the current best evidence of the developed CPG while reflecting their understandability and need for delirium care in LTC.

### 4.3. Limitations

There are some limitations in this study. First, since we only included CPGs published in English or Korean, there could be missing CPGs. Second, in this study, convenience sampling was used for the experts. However, all experts were qualified professors or healthcare professionals in the field with over 10 years of experience who could be expected to successfully evaluate the clinical applicability and feasibility of the developed CPG. Finally, the interviews for identifying barriers to implementation of the CPG were conducted in two LTC settings and all participants were female, so it might not cover all potential barriers and not be transferable to other LTC settings or LTC in other regions.

## 5. Conclusions

Based on the CPG developed in this study, delirium care for older LTC residents in Korea is expected to be improved. Moreover, the developed CPG could also be applied in LTC of other countries with similar contexts, patient populations, and staffing structures to Korea, with minor modifications and specific implementation strategies suitable to the local settings. The developed CPG consists of evidence-based and up-to-date recommendations that reflect the context and resources of general LTC settings. For optimal delirium management in LTC, it is also necessary to implement and continuously update the CPG based on the iterative evaluation of the barriers to implementation by involving users.

## Figures and Tables

**Table 1:** Characteristics of the included CPGs.

Title	Developer	Year	Country	Database Source	Target Patient Population	Quality Score ^a^ (%)
Inter-professional palliative symptom management guidelines	BCPC	2017	Canada	Manual search	adults with any life-limiting illness	40.0
The assessment and treatment of delirium	CCSMH	2014	Canada	G-I-N	older persons	34.38
Care of dying adults in the last days of life	NICE	2015	England	Manual search	adults (≥18) who are dying during their last 2 to 3 days of life	47.11
Delirium: prevention, diagnosis and management (CG115)	NICE	2018	England	G-I-N	adult patients in hospitals or nursing homes	88.08 ^b^
Delirium, dementia, and depression in older adults: assessment and care	RNAO	2016	Canada	G-I-N	older adults (>65)	72.34 ^b^
Risk reduction and management of delirium (SIGN CPG 157)	SIGN	2019	Scotland	G-I-N/Medline	adults	83.22 ^b^

BCPC, British Columbia Center for Palliative Care; CCSMH, Canadian Coalition for Seniors’ Mental Health; EOL, end of life; CPG, clinical practice guideline; G-I-N, Guidelines International Network; NICE, National Institute for Health and Care Excellence; RNAO, Registered Nurses’ Association of Ontario; SIGN, Scottish Intercollegiate Guidelines Network. ^a^ Appraisal of Guidelines for Research and Evaluation II (AGREE II) score. ^b^ Included for development of CPG (total AGREE II score > 50%).

**Table 2:** Recommendations of the developed guidelines.

PICO Questions	Recommendations	LOE
1. What strategy is recommended for preventing delirium in older adults? (1–3)	(1) Assess older adults for delirium risk factors on initial contact and if there is a change in the person’s condition. If any of these delirium risk factors is present, he or she is considered at high risk. [Note] Delirium risk factors: age 65 years or older past or present cognitive impairment, dementia, depression, and/or disorientation severe illness (a clinical condition that is deteriorating or is at risk of deterioration) acute illness and associated abnormal blood values other medical conditions (e.g., infection, fever, dehydration and/or constipation, malnutrition, anemia, hypoxia) sensory deprivation or impairment immobility or limited mobility (e.g., use of physical restraints, prolonged bed rest) sleep deprivation or disturbance poorly controlled pain polypharmacy, use of high-risk medications, or any changes in medications	Ia and V
	(2) Develop and implement a tailored, non-pharmacological, multi-component delirium prevention plan for persons at risk of delirium in collaboration with the person, their families (or care partners), and the interprofessional team, even if the person has not been identified as having delirium. [Note] Possible non-pharmacological interventions for delirium prevention: providing appropriate lighting and clear signage (a clock and a calendar) talking to the person to re-orientate them introducing cognitively stimulating activities (e.g., reminiscence) facilitating regular visits from family and friends looking for and treating infection, avoiding unnecessary catheterization ensuring adequate fluid intake to prevent dehydration by encouraging the person to drink resolving any reversible cause of the impairment, such as removing impacted ear wax affecting hearing and ensuring hearing/visual aids are in good working order encouraging all people, including those unable to walk, to engage in range-of motion activities and to exercise avoiding nursing or medical procedures during sleeping hours, if possible reducing noise to a minimum during sleep periods looking for non-verbal signs of pain, particularly in those with communication difficulties starting and reviewing appropriate pain management in any person in whom pain is identified or suspected	Ia
	(3) All patients at risk of delirium should have a medication review conducted by an experienced healthcare professional, paying particular attention to medications with increased risk for older adults and polypharmacy. [Note] Medications with increased risk for older adults: recently started, stopped, or changed (e.g., doses) medications high-risk medications Benzodiazepines (e.g., diazepam, chlordiazepoxide, clonazepam) Opiates (especially pethidine) others (e.g., antipsychotics, antispasmodics, antiepileptics, antihistamines, antihypertensives, corticosteroids, tricyclic antidepressants, digoxin, antiparkinsonian medication)	Ib
2. What strategy is recommended for early detection of delirium in older adults? (4–8)	(4) Use clinical assessments and validated tools to assess older adults at risk of delirium at least daily (where appropriate) and whenever changes in the person’s cognitive function, perception, physical function, or social behavior are observed or reported. The 4 ‘A’ test (4AT) can be considered for use in identifying older adults with probable delirium.	Ia and V
	(5) Assess older adults at risk for recent (within hours or days) changes or fluctuations in behavior by using a validated tool for delirium detection. Be particularly vigilant for behavior indicating hypoactive delirium (marked*). [Note] These behavior changes may affect: cognitive function: e.g., worsened concentration*, slow responses*, confusion perception: e.g., visual or auditory hallucinations physical function: e.g., reduced mobility*, reduced movement* restlessness, agitation, changes in appetite*, sleep disturbance social behavior: e.g., lack of cooperation with reasonable requests, withdrawal*, or alterations in communication, mood, and/or attitude.	Ia
	(6) Identify and differentiate delirium from the signs and symptoms of dementia, and/or depression during assessments, observations, and interactions with older persons, paying close attention to concerns about changes expressed by the person, his/her family/care partners, and the interprofessional team. If there is difficulty distinguishing between the diagnoses of delirium, dementia or delirium superimposed on dementia, treat for delirium first.	V
	(7) For older adults whose assessments indicate delirium, notify the qualified clinicians (e.g., attending doctors) or refer older adults to the appropriate clinicians, teams, or services for further assessment and diagnosis.	Ia
	(8) When delirium is diagnosed, document clearly in the person’s record and inform the person and his or her family/care partners of the diagnosis. Assess the person’s ability to understand and appreciate information relevant to making decisions and, if the person is incapable of making certain decisions, engage the appropriate substitute decision maker in decision-making and care planning.	V
3. What strategy is recommended for the intervention of delirium in older adults? (9–17)	(9) For older adults whose assessments indicate delirium, systematically identify the possible underlying cause or combination of causes, noting that multiple causes are common. Referring the person for additional investigation can be considered.	Ia
	(10) First consider and treat acute, life-threatening causes of delirium, including low oxygen level, low blood pressure, low glucose level, and drug intoxication or withdrawal. Ensure effective communication and reorientation (e.g., explaining where the person is, who they are, and what your role is).	V
	(11) Implement tailored, multi-component interventions to actively treat the underlying causes, using non-pharmacological means if possible.	Ia and V
	(12) Educate persons who are experiencing delirium and their families/care partners about delirium care and support the person’s ability to make decisions in full or in part.	V
	(13) Although pharmacological treatment is not well supported by evidence, if a person with delirium is distressed or considered a risk to themselves or others and verbal and non-verbal de-escalation techniques are ineffective or inappropriate, consider appropriate use of medications to alleviate the symptoms of delirium. Start at the lowest clinically appropriate dose and titrate cautiously according to symptoms. [Note] Medications for unmanageable agitation/distress: Haloperidol 0.5–1 mg orally (max 2 mg/24 h) Haloperidol 0.5 mg intramuscularly (IM) (max 2 mg/24 h) (* Haloperidol is contra-indicated in combination with the corrected QT interval (QTc) prolonging drugs, which makes it unlicensed and thus local “off label” policy should be followed.) an atypical antipsychotic at low dose, e.g., Risperidone 0.25 mgs daily, maximum 1 mg in 24 h do not use if there are signs of Parkinsonism or Lewy body dementia. If antipsychotics are contra-indicated (as above), Lorazepam 0.5–1 mg orally (max 2 mg/24 h), Midazolam 2.5 mg IM (max 7.5 mg/24 h).	Ia and V
	(14) Use appropriate medications to manage pain.	Ia
	(15) Use the principles of least restraint as a last resort when caring for older adults.	V
	(16) If delirium does not resolve, re-evaluate for underlying causes. Be aware that older people may have pre-existing cognitive impairment that may have been undetected or has become exacerbated in the context of delirium. Appropriate cognitive and functional assessment should be considered. Timing of this assessment must take into account persistent delirium.	V
	(17) Consider referring older adults with delirium to the appropriate clinicians, teams, or services for care.	Ia
LOE Ia. Evidence obtained from meta-analysis or systematic reviews of randomized controlled trials and/or synthesis of multiple studies primarily of quantitative research. Ib. Evidence obtained from at least one randomized controlled trial. IIa. Evidence obtained from at least one well-designed controlled study without randomization. IIb. Evidence obtained from at least one other type of well-designed quasi-experimental study, without randomization III. Synthesis of multiple studies primarily of qualitative research. IV. Evidence obtained from well-designed non-experimental observational studies, such as analytical or descriptive studies and/or qualitative studies. V. Evidence obtained from expert opinion or committee reports and/or clinical experiences of respected authorities.		

LOE, Level of evidence; PICO, Patient Intervention Comparison Outcome.

**Table 3:** The perceived barriers to implementation of CPG for delirium by healthcare professionals.

Theme	Sub-Theme	Quotations
System level		
Lack of resources	Lack of time	“It is difficult because it means that we have to screen (delirium) all 50 people in one day. How much work to do.” P4 “I tried to apply the tool, but there is not enough time to actually use it.” P8 “I think an easier tool or observational tool would be better.” P1
	Lack of education	“We are very confused between delirium and dementia, but it would be easier if there is such information (education) about how this actually appears in the case and how we should screen and manage it.” P1 “It was the first time I have been educated on delirium and the tools. It was interesting that there are tools developed for delirium screening.” P2
	Limited organizational approach	“If it is not compulsory, the guidelines may not be used by those who are not interested.” P6 “I think repetitive education (for delirium) is important. Falls are continuously educated at the facility level, so we naturally can keep in mind and care for them.” P8
Practice level		
Tendency to follow mindlines ^a^ rather than guidelines	I am already knowledgeable	“In fact, (we) know all of the patient’s conditions, so there is a question whether this (delirium care) should be done in LTC. All those who take similar medicines and take similar care in a similar state every time, (there is no need for guidelines).” P5 “Because we are too familiar (to the residents), sometimes something else might be invisible to our eyes. Every day is the same day for us.” P9
	No problems so far	“I have seen little delirium here for many years.” P1 “Most residents are with dementia, so we consider dementia not delirium.” P3 “Even attending doctors diagnose and prescribe focusing more on dementia and BPSD than delirium, and we have been doing quite well.” P2
Healthcare professionals level		
Passive attitude (This is not our job.)		“Delirium treatment is the responsibility of the doctor, not ours. Non-pharmaceutical interventions are some of the things we can do. We just take a step back and look at the patient.” P4 “Even if delirium is observed, we just notify the attending doctor for some drugs or refer the patient to the hospitals.” P7
Patient/family level		
Misunderstanding about delirium care in LTC		“Caregivers don’t want to actively find the cause (of delirium) or treat it. Some caregivers say, ‘Is it necessary?’ when the patient is in the condition requiring additional treatment or drugs.” P4

CPG, clinical practice guideline; LTC, Long-term care; P, Participants of the interviews. ^a^ Mindlines, the usual method or tacit knowledge formed by opinions shared among colleagues.

## Supplementary Materials

The following are available online at https://www.mdpi.com/1660-4601/17/21/8255/s1: Table S1: Predetermined Excel Form for Data Extraction.

## References

[1] American Psychiatric Association (2013). Diagnostic and Statistical Manual of Mental Disorders.

[2] Forsberg M.M. (2017). Delirium update for postacute care and long-term care settings: A narrative review. J. Am. Osteopath. Assoc..

[3] De Lange E., Verhaak P.F., van der Meer K. (2013). Prevalence, presentation and prognosis of delirium in older people in the population, at home and in long term care: A review. Int. J. Geriatr. Psychiatry.

[4] Morichi V., Fedecostante M., Morandi A., Di Santo S.G., Mazzone A., Mossello E., Bo M., Bianchetti A., Rozzini R., Zanetti E. (2018). A point prevalence study of delirium in italian nursing homes. Dement. Geriatr. Cogn. Disord..

[5] Moon K.J., Park H. (2018). Outcomes of patients with delirium in long-term care facilities: A prospective cohort study. J. Gerontol. Nurs..

[6] Reynish E.L., Hapca S.M., De Souza N., Cvoro V., Donnan P.T., Guthrie B. (2017). Epidemiology and outcomes of people with dementia, delirium, and unspecified cognitive impairment in the general hospital: Prospective cohort study of 10,014 admissions. BMC Med..

[7] Brooke J. (2018). Differentiation of delirium, dementia and delirium superimposed on dementia in the older person. Br. J. Nurs..

[8] Marchington K.L., Carrier L., Lawlor P.G. (2012). Delirium masquerading as depression. Palliat. Support. Care.

[9] Cole M.G., Bailey R., Bonnycastle M., McCusker J., Fung S., Ciampi A., Belzile E. (2016). Frequency of full, partial and no recovery from subsyndromal deliriumin older hospital inpatients. Int. J. Geriatr. Psychiatry.

[10] Parrish E. (2019). Delirium superimposed on dementia: Challenges and opportunities. Nurs. Clin. N. Am..

[11] Khurana V., Gambhir I.S., Kishore D. (2011). Evaluation of delirium in elderly: A hospital-based study. Geriatr. Gerontol. Int..

[12] Baker N.D., Taggart H.M., Nivens A., Tillman P. (2015). Delirium: Why are nurses confused?. Medsurg Nurs..

[13] Lima J.C., Intrator O., Wetle T. (2015). Physicians in nursing homes: Effectiveness of physician accountability and communication. J. Am. Med. Dir. Assoc..

[14] Graham I.D., Logan J. (2004). Innovations in knowledge transfer and continuity of care. Can. J. Nurs. Res..

[15] Graham I.D., Logan J., Harrison M.B., Straus S.E., Tetroe J., Caswell W., Robinson N. (2006). Lost in knowledge translation: Time for a map?. J. Contin. Educ. Health Prof..

[16] Brouwers M.C., Kho M.E., Browman G.P., Burgers J.S., Cluzeau F., Feder G., Fervers B., Graham I.D., Grimshaw J., Hanna S.E. (2010). Agree ii: Advancing guideline development, reporting and evaluation in health care. CMAJ.

[17] Registered Nurses’ Association of Ontario (2016). Delirium, Dementia, and Depression in Older Adults: Assessment and Care.

[18] Brouwers M.C., Graham I.D., Hanna S.E., Cameron D.A., Browman G.P. (2004). Clinicians’ assessments of practice guidelines in oncology: The capgo survey. Int. J. Technol. Assess. Health Care.

[19] Nowell L.S., Norris J.M., White D.E., Moules N.J. (2017). Thematic analysis: Striving to meet the trustworthiness criteria. Int. J. Qual. Methods.

[20] National Institute for Health and Care Excellence 2018 Surveillance of Delirium: Prevention, Diagnosis and Management (Nice Guideline cg103). https://www.nice.org.uk/guidance/cg103/resources/2018-surveillance-of-delirium-prevention-diagnosis-and-management-nice-guideline-cg103-pdf-8546233843141.

[21] Scottish Intercollegiate Guidelines Network (SIGN) (2019). Risk Reduction and Management of Delirium (sign 157): A National Clinical Guideline.

[22] Wang Z., Norris S.L., Bero L. (2018). The advantages and limitations of guideline adaptation frameworks. Implement. Sci..

[23] Clegg A., Siddiqi N., Heaven A., Young J., Holt R. (2014). Interventions for preventing delirium in older people in institutional long-term care. Cochrane Database Syst. Rev..

[24] Kolanowski A. (2018). Delirium in people living with dementia: A call for global solutions. Aging Ment. Health.

[25] Cole M.G., Ciampi A., Belzile E., Zhong L. (2009). Persistent delirium in older hospital patients: A systematic review of frequency and prognosis. Age Ageing.

[26] Cole M.G., McCusker J., Voyer P., Monette J., Champoux N., Ciampi A., Belzile E., Vu M. (2014). Core symptoms not meeting criteria for delirium are associated with cognitive and functional impairment and mood and behavior problems in older long-term care residents. Int. Psychogeriatr..

[27] Kiely D.K., Marcantonio E.R., Inouye S.K., Shaffer M.L., Bergmann M.A., Yang F.M., Fearing M.A., Jones R.N. (2009). Persistent delirium predicts greater mortality. J. Am. Geriatr. Soc..

[28] Jeong E., Park J., Lee J. (2020). Diagnostic test accuracy of the Nursing Delirium Screening Scale: A systematic review and meta-analysis. J. Adv. Nurs.

[29] Jeong E., Park J., Lee J. (2020). Diagnostic Test Accuracy of the 4AT for Delirium Detection: A Systematic Review and Meta-Analysis. Int. J. Environ. Res. Pubblic Health.

[30] Hosie A., Lobb E., Agar M., Davidson P.M., Phillips J. (2014). Identifying the barriers and enablers to palliative care nurses’ recognition and assessment of delirium symptoms: A qualitative study. J. Pain Symptom Manag..

[31] Rowley-Conwy G. (2018). Barriers to delirium assessment in the intensive care unit: A literature review. Intensive Crit. Care Nurs..

[32] Brazil K., Royle J.A., Montemuro M., Blythe J., Church A. (2004). Moving to evidence-based practice in long-term care: The role of a best practise resource centre in two long-term care settings. J. Gerontol. Nurs..

[33] Specht J.K. (2013). Evidence based practice in long term care settings. J. Korean Acad. Nurs..

[34] Jeong E., Chang S.O. (2018). Exploring nurses’ recognition of delirium in the elderly by using Q-methodology. Jpn. J. Nurs. Sci..

[35] Pollard C., Fitzgerald M., Ford K. (2015). Delirium: The lived experience of older people who are delirious post-orthopaedic surgery. Int. J. Ment. Health Nurs..

