# Supplementary material for: Development and Evaluation of Clinical Practice Guideline for Delirium in Long-Term Care
Source: Int J Environ Res Public Health. 2020 Nov 9;17(21):8255. doi: 10.3390/ijerph17218255 (PMC7664888; doi:10.3390/ijerph17218255)
Supplement: Supplementary file 1 [file ijerph-17-08255-s001.pdf]

Table S1. Predetermined Excel Form for Data Extraction

General information

NO.	Developer	Title	Publish Date	Country	Publication status	Database source	Language	Target patient population	Full-text (URL)

Recommendations

Categories	Total number	General (Y/N)	Prevention (Y/N)			Early detection (Y/N)				Intervention (Y/N)				
			Risk management	Prevention strategies	Etc	Screening tools	Detection strategies	Lab or imaging	Etc	General	Pharma-cological	Non-pharma-cological	Evaluation/ complications	Etc

Quality assessment

Total score	Domain 1. Scope and Purpose				Domain 2. Stakeholder Involvement				Domain 3. Rigor of Development									Domain 4. Clarity of Presentation				Domain 5. Applicability					Domain 6. Editorial Independence		
	1	2	3	Sub total	4	5	6	Sub total	7	8	9	10	11	12	13	14	Sub total	15	16	17	Sub total	18	19	20	21	Sub total	22	23	Sub total
